# Identification of transcription factor's targets using tissue-specific transcriptomic data in *Arabidopsis thaliana*

**DOI:** 10.1186/1752-0509-4-S2-S2

**Published:** 2010-09-13

**Authors:** Gyan Prakash Srivastava, Ping Li, Jingdong Liu, Dong Xu

**Affiliations:** 1 Computer Science Department and Christopher S. Bond Life Sciences Centre, University of Missouri, Columbia, USA; 2 Monsanto Company, Creve Coeur, St. Louis MO, USA

## Abstract

**Background:**

Transcription factors (TFs) regulate downstream genes in response to environmental stresses in plants. Identification of TF target genes can provide insight on molecular mechanisms of stress response systems, which can lead to practical applications such as engineering crops that thrive in challenging environments. Despite various computational techniques that have been developed for identifying TF targets, it remains a challenge to make best use of available experimental data, especially from time-series transcriptome profiling data, for improving TF target identification.

**Results:**

In this study, we used a novel approach that combined kinetic modelling of gene expression with a statistical meta-analysis to predict targets of 757 TFs using expression data of 14,905 genes in Arabidopsis exposed to different durations and types of abiotic stresses. Using a kinetic model for the time delay between the expression of a TF gene and its potential targets, we shifted a TF's expression profile to make an interacting pair coherent. We found that partitioning the expression data by tissue and developmental stage improved correlation between TFs and their targets. We identified consensus pairs of correlated profiles between a TF and all other genes among partitioned datasets. We applied this approach to predict novel targets of known TFs. Some of these putative targets were validated from the literature, for E2F's targets in particular, while others provide explicit genes as hypotheses for future studies.

**Conclusion:**

Our method provides a general framework for TF target prediction with consideration of the time lag between initiation of a TF and activation of its targets. The framework helps make significant inferences by reducing the effects of independent noises in different experiments and by identifying recurring regulatory relationships under various biological conditions. Our TF target predictions may shed some light on common regulatory networks in abiotic stress responses.

## Background

Plants often respond and adapt to different environmental stresses, such as drought, cold and chemicals through various transcriptional regulatory systems [1]. Identification of these regulations not only enhances our knowledge of biological processes in plants, but also helps a great deal in developing bio-engineered crops that can better sustain challenging environments [2]. Typically, a handful of key transcription factors (TFs) control various biological pathways by regulating downstream target genes. In many cases, these target genes share functions or pathways. While basic ideas of these TFs and their target genes' general functions may be known, lack of knowing explicit target genes often limits the experimental design for validating intuitive hypotheses or developing new crop traits. A comprehensive list of putative targets of a TF could be used to provide more insight of a key TF through functional enrichment analysis or mapping these target genes into different biological pathways.

High-throughput expression profiling experiments [3] have generated large amounts of data that make it possible to develop computational approaches for predicting regulatory relations. Public repositories like NCBI Gene Expression Omnibus (GEO) [4,5], SMD (Stanford Microarray Database) [6], TAIR [7], etc. contain extensive microarray data from time series, developmental stages, genetic interventions or manipulative treatments for *Arabidopsis thaliana*, a model organism for plants [8,9]. These data as well as ChIP-chip data have been used to study interactions of TFs to their downstream genes [10-13]. However, mining microarray data for discovering complicated regulatory relationships is still challenging partially due to the fact that these data are often incomplete, noisy, and contain misleading outliers, all of which likely produce false positives in biological inferences.

Many computational approaches for predicting genome-wide targets of a TF are based on finding co-occurrence of TFs and their targets. These include Standard Pearson correlation technique to measure statistical significance of synchronous co-regulation of genes and order of regulation [14]. However, correlation coefficient is a weak criterion for measuring dependence and can lead to many false positives in predicting TF targets [15]. Another approach is Graphical Gaussian Model (GGM) based on the concept of partial correlation for learning high-dimensional dependency networks from genomic data [16,17], which is valid when number of genes is comparable to number of samples in the microarray data [18,19]. One way to avoid this limitation is to use GGM with regularization and moderation, which is implemented as an R package *GeneNet *[20,21]. This method has been used to infer genome-scale regulatory network for *A. thaliana* transcriptome [22]. Some other methods are based on probabilistic models, such as the Bayesian network [23] and regression tree method [24]. Such methods cannot be directly applied to many time series expression profiling data, because the apparent time lag between initiation of a TF and activation of its targets is not accounted in these models. For example, a study suggests a clear time lag between the mRNA levels of a TF, CBF and its known targets [25]. In part, the time lag is used to translate the mRNAs of a TF into proteins before the proteins can act on activating/repressing TF's targets. To address this issue, it is important to adjust time-series transcription profiling data for detection of TF-target relationship [26].

Another group of methods to identify TF-target genes are specifically designed for time-series expression profiling data, including a method based on Needleman- Wunsch algorithm [27] and a dynamic probabilistic model based on chemical kinetics and linear differential equations [28]. The dynamic probabilistic model, introduced by Friedman et al. [23], is able to learn the kinetic parameters of TFs binding to their target promoters and the structure of gene regulation network simultaneously. However, it requires estimation of a large number of parameters, and it does not provide an explicit way of identifying TFs' targets from predicted active regulator's protein profiles. The linear differential equation model in Ref. [28] describes the production and degradation of all mRNAs and their corresponding proteins with equations of chemical kinetics. While it is an interesting and promising theoretical approach, it tends to be very complex and requires concentration measurements of both mRNA and protein, at least at the initial state.

Many existing studies for retrieving regulatory information use a large collection of microarray data. A potential problem in using microarray data this way is ignoring the heterogeneity in topology of regulatory network due to biological/experimental factors, which could be different tissues, developmental stages or artificial treatments.

**Figure 1:**
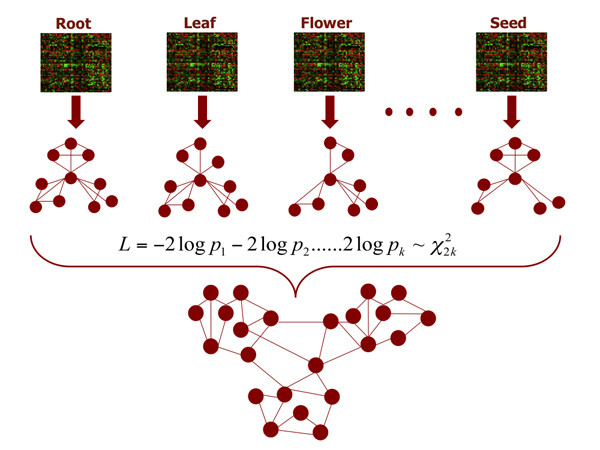
Network construction using meta-analysis of tissue-specific microarray data.

A specific tissue type often has its own set of genes expressed to keep its identity. This may lead to different sets of target genes regulated by the same TF. In our approach, we addressed these issues by performing tissue-wide meta-analysis of expression pattern in at least certain number of tissue types out of all tissue types as shown in Figure 1. In particular, we first perform statistical analysis on microarray datasets of each tissue type and then combine the statistics of multiple microarray datasets for predicting TF targets. Such an approach allows us to identify recurring and stable regulatory relationships under multiple biological conditions while reducing the effects of noises in gene expression data. To avoid the risk of biasing towards housekeeping genes, which are expressed in all tissues all the times, we consider only those genes whose expression profiles are differentially expressed in at least one tissue. The novelty of this approach lies in combining the meta-analysis technique to find consensus regulatory interactions with the kinetic model to estimate the time lag between a TF and its associated targets. The scope of our work is smaller than general regulatory network construction, as we are only interested in recurring targets of known TFs. The reduced scope is practically useful and makes the problem more tractable. We chose the model plant *Arabidopsis Thaliana* for this work given its rich availability of biological data and knowledge.

## Results and discussion

We used a kinetic model combined with statistical meta-analysis to identify TF targets and reconstructed an Arabidopsis global regulatory network using large-scale expression profiles of 14,905 genes. We then evaluated our strategy by comparative and functional analysis of predicted E2F target genes and by comparing our method with other existing methods. Finally, we analyzed the reconstructed network to infer some novel features from the network.

### • Network construction

In order to conduct meta-analysis, we partitioned the datasets based on different attributes including tissue, experiment type and developmental stage. The tissue- specific partition of the microarray datasets produced totally 8 tissue types that have sample size of at least 9. We combined the rest of the samples into one group as combined tissues as shown in Table 1.

**Table 1:** List of all tissue groups used for meta-analysis.

	Tissue Group	Number of Samples	Number of Experiments
1	Seedling	180	9
2	Root	95	14
3	Shoot	68	10
4	Leaf	45	5
5	Flower	33	5
6	Seed	11	3
7	Shoot-apex	10	1
8	Protoplast	9	1
9	Combined rest	46	5

**Figure 2:**
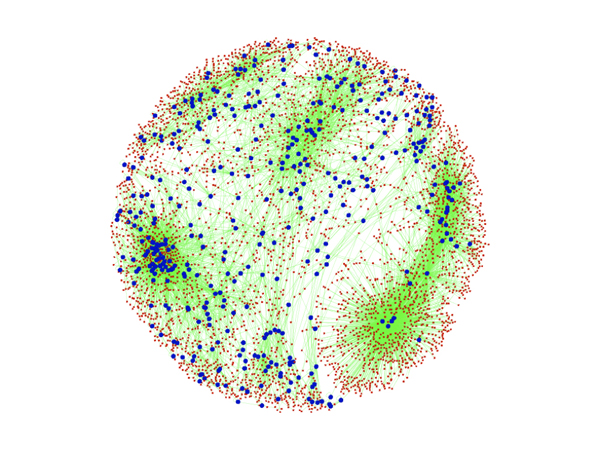
**Global regulatory network with 4968 nodes (genes) and 12,300 edges for Arabidopsis.** Blue (larger) nodes correspond to TFs and red (smaller) nodes correspond to genes that are regulated by TFs. All the edges are marked by green.

We defined the significance level of TF-target pair as number of tissues in which the TF-target pair is significantly co-expressed (p-value < 0.01) after time lag corrections using the kinetic model. We built three networks of ~2K, ~12K and ~59K edges, which correspond to significance levels of more than 9, 8 and 7, respectively. For further analysis, we used the network of ~12K edges to balance the size of network and tolerance of experimental errors in each tissue. This network consists of 12,300 regulatory interactions amongst 4,968 genes, in which 757 genes act as TFs (Figure 2). It is interesting to note that the distribution of the network is highly uneven. In some cases (e.g., lower right), a handful of TFs regulate many putative targets, while in other cases (e.g., left edge) many TFs form clusters among themselves.

### • E2F Network evaluation

In order to assess our TF target prediction with known regulatory mechanisms from the literature, we investigated Arabidopsis E2F family transcription factor "At2g36010", which represents a group of proteins that play a crucial role in the control of cell cycle progression and regulate expression of genes required for the G1/S transition. These include enzymes involved in nucleotide synthesis and DNA replication proteins [29-31]. Though it is clear that E2F is highly critical and conserved amongst high eukaryotes, only a few genes induced by E2F are experimentally verified in plants. Vandepoele et al. [31] combined microarray and promoter motif analyses to identify E2F-targets in plants. To do this, promoter regions of genes that were induced at the transcriptional level in Arabidopsis seedlings were searched for the presence of E2F-binding sites. In another study, Ramirez-Parra et al. [30] identified potential E2F-responsive genes by a genome-wide search of chromosomal sites containing E2F-binding sites. Using meta-analysis of tissue- specific microarray data, we identified 178 putative E2F-target genes (see Additional file 1). Some of these were also predicted by either Vandepoele et al. [31] or Ramirez- Parra et al. [30] as shown in Table 2. As the two other studies used different analytical approaches to identify targets, the overlapping genes among all three methods have more confidence to be true E2F target genes.

**Table 2:** Predicted E2F-target genes from ~12K-size network that overlaps with previous studies by Ramirz-Parra et al. [30] and Vandepoele et al. [31].

	Locus ID	Symbol	Annotation	[30]	[31]
1	At1g08130	ATLIG1	DNA recombination / DNA repair / DNA replication	-	√
2	At1g07370	PCNA1	Regulation of DNA replication and cell cycle	-	√
3	At1g67630	POLA2	DNA synthesis and replication	√	√
4	At2g07690	-	DNA synthesis and replication	√	-
5	At5g66750	CHR1	Transcriptional control/chromatin modification	√	-
6	At1g78650	POLD3	DNA or RNA metabolism/ transferase activity	√	√
7	At4g14700	ORC1A	Cell cycle, Replication control, DNA synthesis	√	-
8	At1g09450	-	N-terminal protein myristoylation/ protein amino acidPhosphorylation	√	-
9	At2g40550	ETG1	DNA replication	√	√
10	At1g67320	-	DNA replication, synthesis of RNA primer	-	√
11	At1g44900	-	DNA synthesis and replication, cell cycle control	√	√
12	At1g69770	CMT3	Chromatin silencing / DNA methylation	-	√
13	At2g21790	RNR1	DNA synthesis and replication	√	√
14	At2g16440	-	DNA replication initiation	-	√
15	At5g38110	ASF1B	Transcriptional control	√	√
16	At5g52950	ATIM	Putative protein	√	-
17	At5g18620	CHR17	Transcriptional control, chromatin modification	√	√
18	At5g52910	ATIM	Regulation of circadian rhythm	√	√
19	At2g24490	RPA2	Replication protein A-like	-	√
20	At2g29570	PCNA2	Error-prone postreplication DNA repair / replication	-	√
21	At2g31270	CDT1A	Chloroplast organization / DNA replication	-	√
22	At3g02820	-	Response to DNA damage stimulus / cell cycle	-	√
23	At3g18630	-	DNA repair	-	√
24	At3g25100	CDC45	Cell division control protein	-	√
25	At5g49010	SLD5	DNA replication initiation / GINS complex	-	√
26	At5g49160	MET1	DNA or RNA metabolism / other cellular processes	-	√
27	At5g62410	SMC2	Cell organization / DNA or RNA metabolism	-	√
28	At5g63960	-	DNA or RNA metabolism / nucleobase, nucleoside, nucleotide and nucleic acid metabolic process	-	√
29	At5g67100	ICU2	Negative regulation of flower development / leaf morphogenesis	-	√
30	At1g35530	-	helicase activity/ hydrolase activity / DNA binding	-	√
31	At3g02920	-	nucleic acid binding	-	√
32	At3g27640	-	nucleotide binding	-	√
33	At5g06590	-	Unknown	-	√
34	At5g63920	-	DNA metabolic process / DNA unwinding duringReplication	-	√

We also conducted functional enrichment analysis for the 178 E2F-target genes identified using meta-analysis. We applied the AmiGO's Term Enrichment tool, which is based on GO-TermFinder [32]. We used all the annotated genes in TAIR [33] as the background set. We selected enriched gene groups with a p-value cutoff of 0.01 and the minimum number of gene products of 2. Our result (Table 3) supports the previous findings that the E2F pathway plays critical roles in cell cycle regulation, DNA replication, and chromatin dynamics. In addition, we identified other novel genes, which are involved in DNA methylation on cytosine, DNA repair, ribosome biogenesis, etc.

**Table 3:** GO term enrichment analysis of 178 predicted E2F-target genes.

GO Term	Description	P-value	Number of Genes
GO:0006260	DNA replication	4.53E-29	23
GO:0006259	DNA metabolic process	1.97E-26	29
GO:0006261	DNA-dependent DNA replication	1.40E-13	12
GO:0006270	DNA replication initiation	6.44E-11	7
GO:0034645	Cellular macromolecule biosynthetic process	2.81E-10	47
GO:0009059	Macromolecule biosynthetic process	3.61E-10	47
GO:0034961	Cellular biopolymer biosynthetic process	7.72E-10	46
GO:0043284	Biopolymer biosynthetic process	9.65E-10	46
GO:0044260	Cellular macromolecule metabolic process	2.13E-09	60
GO:0043170	Macromolecule metabolic process	2.33E-09	61
GO:0034960	Cellular biopolymer metabolic process	3.61E-09	59
GO:0043283	Biopolymer metabolic process	4.82E-09	59
GO:0044249	Cellular biosynthetic process	8.07E-08	50
GO:0044238	Primary metabolic process	1.91E-07	65
GO:0009058	Biosynthetic process	4.48E-07	50
GO:0006139	Nucleobase, nucleoside, nucleotide and nucleic acid metabolic process	6.32E-07	35
GO:0007049	Cell cycle	2.29E-06	12
GO:0044237	Cellular metabolic process	2.88E-06	65
GO:0009987	Cellular process	3.99E-06	77
GO:0008152	Metabolic process	1.64E-05	67
GO:0051052	Regulation of DNA metabolic process	2.18E-04	5
GO:0032776	DNA methylation on cytosine	1.04E-03	3
GO:0006412	Translation	1.80E-03	21
GO:0022402	Cell cycle process	1.98E-03	7
GO:0006281	DNA repair	4.07E-03	7
GO:0034984	Cellular response to DNA damage stimulus	4.29E-03	7
GO:0044267	Cellular protein metabolic process	4.83E-03	31
GO:0019538	Protein metabolic process	5.15E-03	31
GO:0042254	Ribosome biogenesis	5.18E-03	8
GO:0006974	Response to DNA damage stimulus	5.81E-03	7
GO:0022613	Ribonucleoprotein complex biogenesis	5.83E-03	8
GO:0044085	Cellular component biogenesis	8.30E-03	11

### • Network evaluation and comparative analysis

In order to compare performance of meta-analysis with other methods for identifying TF targets, we prepared a benchmark dataset of TF-target pairs in Arabidopsis, which were obtained from the AGRIS database and AtRegNet [34]. The benchmark set has 348 pairs in total. Some of the well-known methods to identify TF target and build regulatory network, including causal regression method, standard Pearson correlation method, and Graphical Gaussian model were used for comparative analysis. To make direct comparison of various methods, we used the exactly same microarray datasets as input to these methods and also exactly the same benchmark data. While using Pearson correlation method, Graphical Gaussian model and regression method, we did not partition the data rather we followed the procedure as previously done in the literature.

In case of microarray data partition and meta-analysis, we used three different ways to partition the microarray data, i.e., tissue based partition, experiment type based partition, and developmental stage based partition. For each type of partition, we identified genome-wide targets for the given set of TFs. While using other methods (Pearson correlation coefficient, causal regression and graphical Gaussian model), we input the microarray data as a single large dataset without partition and identified targets for the same list of TFs. Using these predicted TF-target pairs from each of the methods, we reconstructed two networks of different sizes that is, less than 40,000 edges and less than 70,000 edges. All the same category networks from different methods were then checked against the standard set to count the number of confirmed edges in these networks as shown in Table 4.

**Table 4:** Performance comparison of various methods with Arabidopsis networks of less than 40,000 edges (the numbers before "/") and less than 70,000 edges (the numbers after "/") .

Applied Method		Network Size	Confirmed Edges	Ratio
Pearson Correlation (Cutoff=0.70)		35,253/71,417	25/36	7.09e-4/5.04e-4
Causal Linear Regression Model		30,000/59,557	5/16	1.66e-4/2.68e-4



Graphical Gaussian Model	GeneNet: Static method	30,000/68,624	9/10	3.00e-4/1.46e-4
	GeneNet: Dynamic Method	30,000/68,658	9/10	3.00e-4/1.45e-4



Meta-analysis (Microarray data partition)	Tissue-wide partition	12,300/59,676	35/57	28.5e-4/9.55e-4
	Experiment-wide partition	37,850/56,775	14/18	3.96e-4/3.17e-4
	Development-based partition	37,850/57,339	18/22	4.75e-4/3.84e-4

The results show that our method with partitioning microarray data into tissue- specific datasets and then performing tissue-wide meta-analysis contains the most confirmed edges. Particularly, the network of less than 40,000 edges obtained using tissue-wide meta-analysis is 1/3 in size compared to other networks in the same category, but with more confirmed edges than any other network. The comparison clearly demonstrates that tissue-wide partition performs much better than experiment- wide or development-based partition. This indicates that the gene expression is driven more by tissue types than by development stages and experimental conditions, at least in Arabidopsis. It also shows that the tissue-wide meta-analysis could greatly improve network constructions over other methods. Interestingly, a simple method using Pearson correlation cutoff of 0.70, although not as good as meta-analysis, outperformed sophisticated methods of causal linear regression model and graphical Gaussian model. This may be because microarray data are often noisy and sophisticated methods could amplify noises to give incorrect predictions in gene regulatory relationships.

### • Network feature analysis

**Figure 3:**
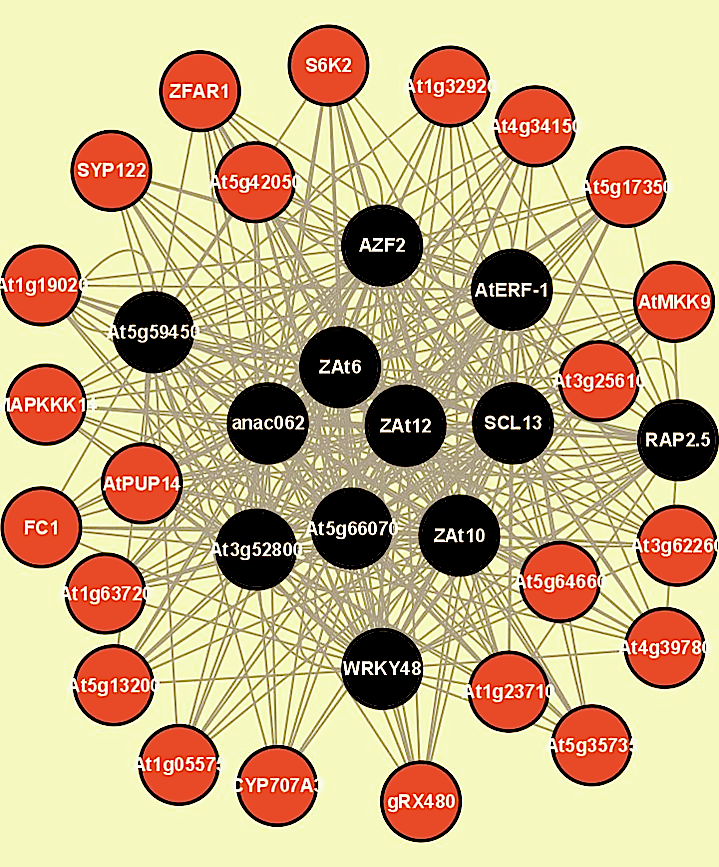
**A cluster with hub genes identified using MCODE.** This sub-network contains 35 nodes and 362 edges. Black nodes indicate TFs and red nodes indicate target genes.

Using Cytoscape [35], we identified a few major hubs (nodes with many connections) from the medium sized network (~12K) using tissue-wide meta-analysis. In particular, we found regions of significant local density using the MCODE plugin [36] from Cytoscape. Figure 3 shows an example of a major hub cluster, which represents 12 TFs including SCL13, ZAT6, AtERF-1 and anac062 each targeting many genes as found in Table 5 from further analysis.

**Table 5:** Global regulators from medium size network having most target genes. A complete list of putative targets for each TF is available in Additional file 2.

	Locus ID	Symbol	Annotation	Target
1	AT2G38470	WRKY33	Response to drought, heat, chitin, osmotic stress, salt, cold etc., defense response to fungus, bacterium	216
2	AT1G80840	WRKY40	Response to wounding, salicylic acid, chitin, defense response to bacterium, fungus etc	102
3	AT3G49530	anac062	Response to chitin	130
4	AT3G57150	NAP57	Pseudouridine synthesis	322
5	AT4G37490	CYCB1	Response to gamma radiation, regulation of cell growth	168
6	AT3G22780	TSO1	Regulation of meristem organization	134
7	AT4G17500	AtERF-1	Response to chitin, regulation of transcription, DNA- dependent	120
8	AT4G30930	NFD1	Embryo sac & pollen development, karyogamy, double fertilization forming a zygote and endosperm	518
9	AT5G59820	RHL41	Response to chitin, heat, UV-B, wounding, oxidative stress, cold, photosynthesis, hyperosmatic salinity response	122
10	AT4G17230	SCL13	Response to chitin	121
11	AT5G04340	ZAT6	Nucleic acid & zinc ion binding, transcription factor activity	139
12	AT1G27730	STZ	Response to abscisic acid, drought, light, cold, chitin, salt etc	128

Beside network feature analysis using Cytoscape, we analyzed TFs that target significantly more genes than other TFs across different tissues as shown in Table 5. Not surprisingly, most of these TFs are annotated with response to different stimulus in Arabidopsis, such as response to chitin and external stress, given that the microarray data we used were measured in response of Arabidopsis exposed to different abiotic stresses. Some of well-connected TFs are also present in hubs as recognized by the MCODE plugin from Cytoscape and are known to work together for gene regulation. For example, Zat6, Zat10, and Zat12 in the hub of Fig. 3 are activated together in cold and osmotic stresses [37]. WRKY33 and WRKY40 in Table 5 both function as activators of jasmonic acid-dependent defence pathways and repressors of salicylic acid signalling [38].

## Conclusion

In this paper, we proposed a meta-analysis method for identifying TF targets. The novelty of the proposed method lies in combining two models that is (1) adjusting time lag between a TF and its target and (2) finding consensus regulatory interactions from different experimental sources/conditions including tissue types, developmental stages and experimental settings. Our study shows that tissue-wide partition performs much better than experiment-wide or development-based partition for predicting TF targets. The method successfully identified more known TF-target pairs in Arabidopsis than other methods.

There are some limitations of this study. Like other approaches, our method has both false positives and false negatives in predictions. Without performing a large-scale experimental validation, there is no reliable way to assess the prediction accuracies of the methods like ours. Hence, the value of our study is mainly to provide hypotheses for experimentalists to explore specific gene regulations of their interest, especially as most of the predicted TF targets with high confidence were not reported or predicted previously. Furthermore, our method may not be able to distinguish TF targets from other co-expressed non-target genes, although meta-analysis across multiple tissues reduces such a possibility. From the meta-analysis point of view, tissue-wide meta- analysis does not consider specific regulatory relations in particular tissue types. In plant, some regulations are specific to different tissue types or developmental stages. Since such relations do not exhibit significant correlation across different microarray data, meta-analysis may ignore them. Nevertheless, meta-analysis is more robust to find correlations that are consistent across different tissues. Typically, global regulations are those that are fundamental for the existence of all tissues in general.

In the context of our study, we only applied gene expression data of Arabidopsis exposed to different abiotic stresses. It is known that there are common regulatory mechanisms for abiotic stresses. For example, certain heat-shock proteins are commonly elicited in response to various stress conditions in multiple plants [39]. Conserved regulatory mechanisms among responses to drought, salinity, and extreme temperature in Arabidopsis were identified, such as the DREB transcription factors [40]. Characterizing common gene expression patterns under various abiotic stress conditions in plants can help elucidate these conserved regulatory mechanisms [41]. Hence, the meta-analysis that we provided on gene expression data under different abiotic stress treatments may shed some light on common regulatory networks in abiotic stress responses. In our future studies, we will explore more into meta-analysis of microarray data by applying different statistics like meta correlation instead of chi- square statistics. Another dimension of improvement is to include inferences from other types of data such as promoter motif analysis.

## Materials and methods

### •	Data preparation

We used publically available microarray data of *A. thaliana* from NCBI GEO (http://www.ncbi.nlm.nih.gov/geo/) and TAIR (http://www.arabidopsis.org/). The microarray gene expression data were normalized and preprocessed in the databases. We removed genes with missing expression measurement in any tissue type and averaged the replicated expression data. Consequently, we applied our method on 497 arrays in total measuring whole-genome response of Arabidopsis exposed to different durations and types of abiotic stresses. Some 14,905 genes from Arabidopsis genome including 757 TFs were chosen for the analysis as each of these genes was differentially expressed in at least one of the stress conditions. The datasets consist of 27 different microarray experiments, out of which 10 experiments are time series (see Additional file 3).

### •	Chemical kinetics models to identify regulator-target relationships

**Figure 4:**
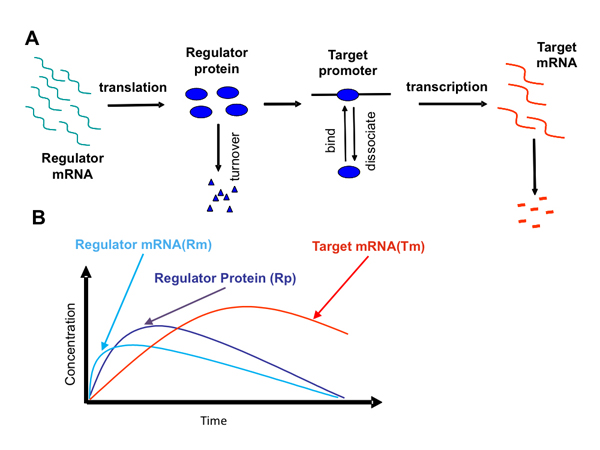
**Schematics of transcriptional regulation process.** A. Steps of chemical reactions considered in the kinetics model. B. Schematics of the temporal curves of regulator protein and target mRNA in response to the regulator mRNA changes.

In eukaryotic cells, the effect of a regulator is usually achieved in multiple steps, including transcription of the regulator genes, transportation of the regulator mRNA(s) out of the nucleus, translation of the transcript(s), transportation of the regulator protein back to the nucleus, and the binding of the regulator protein to the promoter regions of its target genes to achieve transcriptional regulation. Noticeable timing difference exists among changes in concentrations of the regulator mRNA, the regulator protein, and the mRNAs of its targets. A chemical kinetics model naturally fits this context by taking into account of the time lags among these events (Figure 4).

Because the active level of the regulator protein is not measured directly in microarray experiments, the regulator protein concentration is treated as a hidden variable in our model to serve as the link between the measurable mRNA concentrations of a regulator and its target(s). More specifically, the regulator protein concentration can be modelled by the following chemical kinetic equation without considering post-translational regulation:

 (1)

where *R_P_* is the regulator protein concentration, *R_m_* is the regulator mRNA concentration, *K_tram_* is the apparent rate of mRNA translation, and *K_P_* is the turnover rate of the regulator protein. Accordingly, the time course of the target mRNA concentration can be modelled with the following equation

	(2)

where *T_m_* is the concentration of the target mRNA; *B_t_* is the basal transcription rate of the target gene; and *K_t_* is the turnover rate of the target mRNA; *f (R_P_)* measures the regulated transcription rate, which is different for activators and repressors. For activators, it has the following Taylor first order approximation when *R_P_* is small [28].

	(3)

*f* (*Rp*= 0) is equal to zero, assuming target gene transcription should not be activated when there is no regulator protein. is the activation rate of regulator protein on the target gene. If it is replaced by parameter *K_act_* for simplicity, *f* (*R_P_*) takes the following form:

*f* (*R_p_*) = *K_act_R_p_*.	(4)

The basal level target transcription rate should satisfy the following condition:

*B_t_* + *f* (*R_pbasal_*) — *K_t_T_mbasal_* = 0                                  	(5)

where *R_pbasal_* and *T_mbasal_* are the basal concentrations of the regulator protein and target mRNA, respectively.

Usually, what is reported in transcription profiling experiment is not the absolute concentration of mRNA, but rather a fold change compared to basal transcription level of that gene. Thus, we define relative changes of *R_m_* and *T_m_* as *R_m_*' and *T_m_*'

*R_m_*' = *R_m_* ⁄ *R_mbasal_* — 1 ;	(6)

*T_m_*' = *T_m_* ⁄ *T_mbasal_* — 1 .	(7)

Combining Equations (1), (2), (4), (5), (6) and (7), and considering the fact that *K_tran_R_mbasel_ - K_p_R_pbaseal_* = 0, lead to the following second order ordinary differential equation:

	(8)

where *γ* = *K_act_K_tran_R_mbasal_* ⁄ *T_mbasel_* .

Given all the model parameters, the relationship between the relative mRNA levels of the regulator and its target, *R_m_*' and *T_m_*', is defined by Equation (8). In other words, for a target gene of a regulator, its relative mRNA level *T_m_*' has to satisfy Equation (8), given the model parameters and the relative regulator mRNA level *R_m_*'. It is interesting to note that the regulator protein concentration, a key variable in the original model equations, is not involved explicitly in the final equation relating the relative mRNA levels of regulator and target. To predict the target of a specific regulator, we can solve Equation (8) to obtain the theoretical target behaviour curve, and then find the genes with mRNA levels similar to the theoretical curve, which will be identified as the potential targets of that regulator.

In the case of transcript expression profiling experiments under stress conditions, the initial conditions should be the following:

	(9)

	(10)

Because the target gene mRNA and the regulator protein should be at their basal levels at the onset of stress condition (t=0). It is apparent from Equations (2) and (5) that initial condition (10) should be true.

To approximate *R_m_* , a stepwise linear model can be fit as follows:

*R_m_*'*_I_* (*t*) = *α _i_* + *β_i_tt_i_* ≤ *t* ≤*t_i_*_ + 1  _*i* = 0, ··· ,*n*-1    	(11)

where *t_i_* is *i ^th^* time point; and *α_i_* and *β_i_* are the parameters of stepwise linear function in each time interval, which are determined by the measured regulator mRNA levels at the two adjacent time points. Equation (8) has analytic solution

	(12)

Where *D_i_* = *β_i_γ*/*K_p_K_t_* and *Ci* = [*α_i_γ* – (*K_p_* + *K_t_*)*D_i_*]/ *K_p_K_t_* .

The contiguous restrictions on *T_m_*' are stated in the following equations:

*T_mi_*' (*t*) = *T*_*mi* + 1_' (*t*),    where  *t* = *t_i_  i* = 1,…, *n* - 1.	(13)

(14)

After substituting Equation (12) into Equations (9), (10), (13) and (14), *A_i_* and *B_i_* can be obtained by solving sets of linear algebra equations, and are functions of *α_i_*, *ß_i_*, *γ*, *K_t_* and *K_P_*.

#### Learning model parameters and transforming the time series profiles of transcription factors.

For each regulator-target pair, there are three parameters involved in Equation (8), the target mRNA turnover rate *K_t _*, the active regulator turnover rate *K_P_*, and *γ*, which is equal to *K_act_K_trαn_R_mbαsl_ / T_mbαsal_ . K_αct_* represents the strength of regulator protein effect on the target gene; *K_tram_* is the translation rate of regulator mRNA. They lump together with the ratio of basal mRNA concentrations of regulator and target to form parameter γ, which determines the magnitude of the relative target mRNA level but not its shape. It is the parameters *K_t_* and *K_P_* that determine the shape of the relative target mRNA level, such as how fast the target gene responds to the regulator. For gene expression experiments under stress conditions in plants, the kinetics model can be trained with known regulator-target pair reported in the literature (e.g., CBF and RD17 in *Arabidopsis* under cold stress) with a non-linear regression model [42]. When the normalized expression profile of a target gene with its maximal response is considered, there is no need to keep *γ* as a free model parameter (*γ*_1_ = *nγ*_2_ leads to *T*_*m*1_'= n*T*_*m*2_' when other parameters are kept the same in Equations (8), (9) and (10)). Therefore, only two parameters *K_t_* and *K_P_* are estimated from the non-linear regression model, and are used to predict other regulators and their targets in plant stress response.

The theoretical TF-target mRNA expression profiles are calculated for all the genes annotated as TFs and are substituted in place of TFs' profiles during further computation for co-expression calculation. The theoretical target profile of any TF in terms of relative expression levels among different time points is independent of actual targets of that TF as it is solely calculated based on the kinetic model. According to the model, the theoretical target profile of a TF should match the profile of its actual targets in the trend of expression although not in the absolute abundance. With this assumption, we can use Pearson correlation coefficient to find similarity of co-expression between the theoretical/shifted profile of a TF and rest of the genes to find potential targets of this TF.

### • Co-expression statistics

We used a statistical meta-analysis technique [43] to identify highly correlated expression profiles from multiple microarray datasets. Using this technique, we evaluated the statistical significance (right-tailed p-value) of a Pearson correlation coefficient r for two expression profiles in a single dataset based on the standard t- statistics:

	 (15)

where *T* is a *t*–random variable with *n-2* degree of freedom and *n* is the number of conditions of the gene expression profiles. Since we assume that the datasets are obtained independently, we apply the inverse chi-square method and obtain the meta chi-square statistics:

	(16)

where *P_i_* is the p-value obtained from the *i ^th^* data set for a given gene pair defined in Equation (15). When there is no linear correlation between a gene pair in any of the multiple datasets, the above chi-square statistics  follows a central chi-square distribution with degrees of freedom *2n* and hence the p-value for meta-analysis can be obtained by

	(17)

where  is a chi-square random variable with *2n* degrees of freedom. We calculate significance level of the gene pair from multiple datasets. The significance level of gene pair represents the count of datasets in which that gene pair has significant correlation (p-value < 0.01) based on Equation (15). We used meta p-value statistics (Equation (17)) combined with significance level to rank potential targets for a TF [43].

### • Regulatory network reconstruction

The meta p-value combined with significance level and the Pearson correlation coefficient were used as co-expression statistics for finding putative targets for a TF. For a single dataset (without partitioning of microarray data), we ranked all the potential targets of a TF based on Pearson correlation coefficient and select targets such that TF-target correlation > 0.75 (medium size network) or 0.70 (large size network). For multiple datasets, we ranked all TF-target pairs based on the number of individual p-values that are smaller than 0.01 across multiple datasets; for pairs that have the same number of significant p-values, they were ranked by the corresponding meta chi-square statistics defined in Equation (16). Here we used meta chi-square instead of meta p-value since the meta p-value for many gene pairs are very close to zero and hard to distinguish computationally; both meta chi-square and meta p-value should result in the same order when the degrees of freedom for each gene pair is same. In the end, a fixed number of TF-target pairs were selected based on ranking.

In case of meta-analysis, number of target genes for a TF was determined in two methods, i.e., (1) selecting fixed number of targets from top (50 or 75) or (2) choosing targets form top-ranked genes that shows significance correlation as TF-target pair in at least certain number of microarray datasets used for meta-analysis. For example, we used significance cutoff 9 (out of 9 datasets) for small network and cutoff 8 (out of 9) for medium network and cutoff 7 (out of 9) for large network. The second method worked better in general.

## Competing interests

The authors declare that they have no competing interests.

## Authors' contributions

JL and PL conceived the initial study and prepared relevant data and their preprocessing. GPS and DX designed the statistical method. PL and JL implemented kinetic model. GPS and DX performed the data analyses. All wrote the manuscript.

## Supplementary Material

Additional file 1 A list of identified 178 putative E2F-target genes. Click here for file

Additional file 2 A complete list of putative targets for each TF. Click here for file

Additional file 3 A list of 27 different microarray experiments, out of which 10 experiments are time series. Click here for file
